# Supplementary material for: Dynamic Shape Modulation of Deflated and Adhered Lipid Vesicles
Source: J Am Chem Soc. 2025 Oct 10;147(42):38720–31. doi: 10.1021/jacs.5c13925 (PMC12550850; doi:10.1021/jacs.5c13925)
Supplement: Supplementary file 1 [file ja5c13925_si_001.pdf]

Supporting Information: Dynamic Shape Modulation of Deflated and Adhered Lipid Vesicles

Gianna Wolfisberg,[†] Jaime Agudo-Canalejo,[‡] Pablo C. Bittmann,[†] Eric R. Dufresne,[†] Robert W. Style,[†] and Aleksander A. Rebane[*,†,¶]

†*Department of Materials, ETH Zürich, 8093 Zürich, Switzerland.*

‡*Department of Physics and Astronomy, University College London, London WC1E 6BT, United Kingdom*

¶*Life Molecules and Materials Lab, Programs in Chemistry and in Physics, New York University Abu Dhabi, P.O. Box 129188, Abu Dhabi, United Arab Emirates.*

E-mail: a.rebane@nyu.edu

Materials and Methods

GUV Preparation

Giant unilamellar vesicles (GUVs) were made by electroformation, following the protocol from Ref. 1, on indium tin oxide (ITO) coated glass plates (MSE Supplies, 10x10x1.1 mm with a sheet resistance of 3-5 Ohm/Sq (ITO layer thickness of 350 nm)).[1–5] We used a lipid composition of 99.9% (mol/mol) 1-palmitoyl-2-oleoyl-glycero-3- phosphocholine (POPC) (Avanti Polar Lipids, 850457C) and 0.1% (mol/mol) of 1,2-dioleoyl-sn-glycero3-phosphoethanolamine-N-(lissamine rhodamine B sulfonyl) (ammonium salt) (DOPE-Rh) (Avanti Polar Lipids, in

chloroform, 810150C).

The electroformation chamber consisted of two ITO-coated glass plates separated by a 3 mm polydimethylsiloxane (PDMS) (Sigma Aldrich, Sylgard 184) spacer with cut-out wells of a 9 mm radius and small channels for filling. The lipid solutions were diluted in chloroform (Sigma Aldrich, 132950) to a final lipid concentration of 1 mM. We added dropwise 10 µL of the lipid solution to each well and let the chloroform evaporate. The residual chloroform was evaporated under vacuum overnight. The chamber was closed with the second ITO-coated glass plate, secured with clamps, and filled with GUV buffer. The GUV buffer consists of a filtered (TPP, 0.22 µm PES membrane) sucrose (Sigma Aldrich, S7903) solution at 25 mOsm/kg (GUV buffer), as determined by a freezing point osmometer (Osmomat 3000, Gonotec).

A function generator (Keysight, 33210A) was attached to the ITO plates through aluminium strips each side, resulting in an electric field perpendicular to the ITO plates. The AC voltage was increased in steps of 5 minutes to 5 V (0.83 V, 1.66 V, 2.5 V, 3.3 V, 4.1 V, 5 V) at a constant 10 Hz. The field was then kept for two hours at 5 V and 10 Hz and finally for 30 minutes at 5 V and 5 Hz. The vesicles were removed with glass pipettes and stored in glass tubes at 4° C, where they remained stable for weeks.

SUV and SLB Preparation

Supported lipid bilayers (SLB) were made by fusing small unilamellar vesicles (SUVs) onto glass coverslips (VWR, No. 1.5).[6] For the SUVs, 25 µL of 30 mM lipids with 99.9% (mol/mol) POPC (Avanti Polar Lipids, 850457C) and 0.1% (mol/mol) of 1,2-dioleoyl-sn-glycero-3-phosphoethanolamine-N-dibenzocyclooctyl (DOPE-DBCO) (Avanti Polar Lipids, 870129C) were dried to a film in a glass test tube under flushing argon gas. Residual chloroform was evaporated under vacuum overnight. The lipids were then resuspended in 1 mL SUV buffer by pipetting until the liquid was turbid. The SUV buffer consisted of 25 mM HEPES (Sigma Aldrich, H3375), 140 mM KCl (VWR, 26764.232), and Alexa Fluor 647

azide triethylammonium salt (Invitrogen, A10277) at a 1:1 molar ratio to the DOPE-DBCO lipids. The buffer was adjusted to pH 7.4 and filtered (TPP, 0.22 µm PES membrane). The solution was tip-sonicated (Branson, Sonifier 250) for 2-3 minutes at power 2 and output 20 % for 5-10 cycles with breaks of 5 minutes until the solution became transparent, taking care to avoid significant heating of the solution. Finally, the solution was centrifuged (Eppendorf, 5418 R) for 5 minutes at 16'000 g and the supernatant with SUVs was collected and stored at 4° C until used.

The glass coverslips were cleaned by immersing them in water and treating them in a bath sonicator (Fisherbrand, FB11201) for 10 minutes at 37 kHz at 100%. This was followed by immersion and sonication of the coverslips in ethanol. Next, the coverslips were rinsed with water, blow-dried with air, and surface-activated in a UV/Ozone cleaner (Bioforce Nanoscience, UV/Ozone Cleaner ProPlus) for 10 minutes. Immediately after UV/Ozone treatment, imaging spacers (SecureSeal, GRACE bio-labs, SS1X9) with 9 mm diameter and 120 µm depth were stuck onto the coverslips and the SLB was formed by adding 30-40 µL of SUV solution diluted 1:20 with SLB buffer to the spacer annulus. The SLB buffer consisted of 10 mM Tris pH 7.5 (Thermo Scientific, J63831), 150 mM NaCl (VWR, 27810.295), 2 mM $CaCl_2$ (Sigma Aldrich, 902179) in water and was filtered (TPP, 0.22 µm PES membrane) prior to use. This coverslip and spacer containing the SUV solution was incubated overnight and stored in a humidity box until used. Just before the experiment, residual SUV solution was removed by washing the SLB with 3 mL of outside buffer (10 mM NaCl (VWR, 27810.295) and 5 mM glucose (Sigma Aldrich, G7528)) and then with 3 mL outside buffer including 100 kDa polyethylene glycol (PEG) (Sigma Aldrich, 181986) at the final concentration of 0.2% (w/v), 0.4% (w/v) or 0.8% (w/v). The PEG concentration after filtration (TPP, 0.22 µm PES membrane) was determined by ^{1}H NMR spectroscopy. Deuterated water (D_2O) (Apollo Scientific, DE50B) was used as the solvent and dimethylformamide (DMF) (Fisher Scientific, D/3840/17) was used as a concentration standard as previously described.[7] The PEG concentration was determined based on the ratio of the integrated peaks corresponding

to DMF (at known concentration) and the PEG.

Diffusion Chamber for Deflation at Constant Adhesion Strength

The SLB covered with PEG-containing outside buffer was placed on the microscope and 1 or 10 µL GUV solution were slowly added, subsequently letting the GUVs sediment for 5 to 20 minutes. Meanwhile, a dialysis membrane (3.5 kDa Mini Dialysis Device 2 mL, SlideA-Lyzer) was washed with water and equilibrated with outside buffer. The empty dialysis membrane was then carefully placed onto the spacer and 2 mL of outside buffer were added inside the dialysis button. The dialysis membrane was permeable to water, glucose, and NaCl, but not to PEG 100 kDa.

The system was left to equilibrate for 1 hour. Meanwhile, 10-15 microscope stage positions were selected where vesicles were visible, and confocal z-stacks were acquired (z-step size 0.27 µm) using a spinning disc confocal microscope (Nikon Eclipse Ti2 base with Yokogawa CU-W1 with XYZ automated stage with piezo Z-axis PZ-2300 from Applied Scientific Instrumentation) on a 60x oil objective (Nikon, NA 1.4). The vesicle and SLB membranes were imaged with the 560 nm and 640 nm excitation laser lines, respectively. After imaging of each deflation step, 50 µL of the solution inside the dialysis cup was extracted and its osmolarity was measured using a freezing point osmometer (Osmomat 3000, Gonotec). To achieve a homogeneous concentration in the dialysis membrane, around 800 µL solution was removed, externally mixed with 50 uL deflation solution, added back, and mixed by pipetting up and down. At each deflation step, the composition and osmolarity of our system equlibrated within an hour via diffusion across the dialysis membrane. To obtain the desired resolution of different shapes through deflation, this process was repeated 4 times, while we increased the amount of added deflation buffer from 50 µL to 100 µL, 100 µL, and 200 µL. The total volume in the dialysis cup was kept constant at 2 mL.

In total, each experiment lasted at least 4 h due to ∼1 h for solute equilibration per deflation step. We typically identified ∼15 defect-free GUVs per experiment and followed

them through the deflation series, ∼60 deflation series across three PEG concentrations. After excluding vesicles with tubules or other defect, our final dataset comprised $N = 21$ series (∼5 per experiment). The complete dataset for all GUVs used in our analysis is shown in Figs. S6 - S8.

Image Processing

The SLB and GUV adhesion zone are in the xy-plane. The stacks are sliced in xz to obtain an orthogonal sliced vesicle cross-section. We correct for a z-aberration from the use of an oil-immersion objective by a factor of 0.878, resulting in a z-step of 0.237. First, we identify single GUVs over all time points by location. Then the vesicle is vertically sliced through the center into 3 orthogonal xz cross-sections. With the 3 slices, we estimate the deviation from perfect radial symmetry and create error bars.

Shape Fitting with Canham-Helfrich Model

The shapes and accompanying mechanical parameters were found by solving the Canham-Helfrich model with adhesion following previously described procedures.[8,9] Only axisymmetric shapes were considered. These shapes could therefore be described as surfaces of revolution around the z-axis of a contour parametrized by the arc-length s. The contour was determined by the coordinates perpendicular and parallel to the axis of symmetry, $(X(s),\, Z(s))$ with the accompanying geometric relations (Fig. S15),

$$\frac{\mathrm{d}X}{\mathrm{d}s} = \cos\psi \tag{S1}$$

$$\frac{\mathrm{d}Z}{\mathrm{d}s} = -\sin\psi \tag{S2}$$

$$C_1 = \frac{\mathrm{d}\psi}{\mathrm{d}s} \tag{S3}$$

$$C_2 = \frac{\sin\psi}{X}, \tag{S4}$$

where ψ is the tilt angle of the surface with respect to the x-axis, and C_1 and C_2 are the principal curvatures. The vesicle shape minimizes the free energy,

$$F = \underbrace{\frac{\kappa}{2}\oint (C_1 + C_2)^2 \,\mathrm{d}A}_{E_{\text{bending}}} + \underbrace{\sigma A}_{E_{\text{tension}}} + \underbrace{\Delta P \cdot V}_{E_{\text{pressure}}} - \underbrace{\omega A_{adh}}_{E_{\text{adhesion}}}, \tag{S5}$$

where κ is the bending rigidity, σ is the membrane tension, A is the membrane surface area, $\Delta P = P_{in} - P_{out}$ is the vesicle pressure, V is the volume of the vesicle, ω is the adhesion strength, and A_{adh} is the adhered area. Using the parametrization defined by Eqs. S1-S4 yields the energy functional

$$F\left[X(s), \psi(s), s\right] = 2\pi \int_0^{s_1} L\left(X, \dot{X}, \psi, \dot{\psi}, \gamma\right) \,\mathrm{d}s, \tag{S6}$$

where $L\left(X, \dot{X}, \psi, \dot{\psi}, \gamma\right)$ is the Lagrange function

$$L\left(X, \dot{X}, \psi, \dot{\psi}, \gamma\right) = \frac{\kappa}{2}\left(\frac{\mathrm{d}\psi}{\mathrm{d}s} + \frac{\sin\psi}{X}\right)^2 X + \sigma X \,\mathrm{d}s + \frac{PX^2}{2}\sin\psi + \gamma \cdot \left(\frac{\mathrm{d}X}{\mathrm{d}s} - \cos\psi\right) \tag{S7}$$

and s_1 is the arc length at which the shape meets the surface with

$$\psi(s_1) = \pi \tag{S8}$$

as shown in Fig. S15, and $\gamma = \gamma(s)$ is a Lagrange parameter function to enforce the geometric constraint, $\dot{X} = \cos\psi$. This parametrization divides the shape into a free portion (Fig. 5 A-C *green*), which is derived from numerical integration, and a flat adhered portion (*blue*), which is defined implicitly as a disc with radius $X(s_1)$. It is useful to adopt the non-dimensionalized variables, $\overline{X} = X \cdot U_0$, $\overline{Z} = Z \cdot U_0$, and $\overline{s} = s \cdot U_0$, where $U = \dot{\psi}$ is the principal curvature curvature along the contour, C_1, and $U_0 = |U(s = 0)| = 1/|R_{top}|$, with R_{top} the signed radius of curvature at the top of the vesicle. Minimization of the energy functional, F (Eq. S6),

yields the non-dimensionalized Euler-Lagrange equations,

$$\frac{\mathrm{d}\psi}{\mathrm{d}\overline{s}} = \overline{U} \tag{S9}$$

$$\frac{\mathrm{d}\overline{U}}{\mathrm{d}\overline{s}} = -\frac{\overline{U}}{\overline{X}}\cos(\psi) + \frac{\cos(\psi)\sin(\psi)}{\overline{X}^2} + \frac{\overline{\gamma}}{\overline{X}}\sin(\psi) + \frac{\Delta\overline{P}\,\overline{X}}{2}\cos(\psi) \tag{S10}$$

$$\frac{\mathrm{d}\overline{\gamma}}{\mathrm{d}\overline{s}} = \frac{\overline{U}^2}{2} - \frac{\sin^2(\psi)}{2\overline{X}^2} + \Delta\overline{P}\cdot\overline{X}\sin(\psi) + \overline{\sigma}, \tag{S11}$$

and

$$\frac{\mathrm{d}\overline{X}}{\mathrm{d}\overline{s}} = \cos(\psi), \tag{S12}$$

We then numerically solve these equations using initial conditions

$$\overline{X}(\overline{s}=0) = 0 \tag{S13}$$

$$\psi(\overline{s}=0) = 0 \tag{S14}$$

$$\overline{\gamma}(\overline{s}=0) = 0 \tag{S15}$$

and

$$\overline{U}_0 = \overline{U}(\overline{s}=0) = \pm 1\,, \tag{S16}$$

whereby the last equation defines the direction of the curvature, with $\overline{U}_0 = 1$ for convex and $\overline{U}_0 = -1$ for concave vesicle tops, respectively. The three parameters that control the shape of the vesicle are the normalized tension $\overline{\sigma} = \sigma \cdot R_{top}^2/\kappa$, the normalized pressure $\Delta\overline{P} = \Delta P \cdot R_{top}^3/\kappa$, and the initial condition of convex or concave $\overline{U}_0$ (Eq. S16).

The equations were solved using the ode45 function in MATLAB and by integrating from the top of the vesicle until s_1 defined by Eq. S8. The adhesion strength, was determined from the curvature at the contact line (Fig. 2A),[9]

$$\frac{1}{\overline{R}_c} = \dot{\psi}\left(\overline{s}_1\right) = \sqrt{2\overline{\omega}}\,. \tag{S17}$$

The dimensionful mechanical parameters were obtained by using $\kappa = 33\,\mathrm{k_B T}$ for POPC membranes and the best-fit U_0.[1]

The calculated curves were overlaid on the experimentally observed radial slices using a custom MATLAB GUI. First, the axes of symmetry of the experimentally observed vesicle and the numerically calculated shape were aligned. Next, the maximum radius, R_{max} (Fig. 2A), of the numerically calculated shape was adjusted so that maximum widths of the calculated shape and the observed shape coincided.

Vesicles approximating spherical caps (Fig. 5A) were fitted with convex top curvature, $\overline{U}_0 = 1$, and overpressure, $\Delta\overline{P} > 0$. A ratio $\Delta\overline{P}/\overline{\sigma} = 2 - \varepsilon$, with $\varepsilon << 1$, yielded spherical cap-like shapes, whereby greater absolute values of $\overline{\sigma}$ and $\Delta\overline{P}$ decreased the reduced volume and contact angle of the shape (see S16). Decreasing the ratio $\Delta\overline{P}/\overline{\sigma}$ increasingly flattened the shapes, culminating in perfectly flat shapes for $\Delta\overline{P} = 0$. Accordingly, flat disc-like shapes were fitted with a convex top curvature with $\Delta\overline{P} \approx 0$ (Fig. 5B). However, concave top curvature and $\Delta\overline{P} \ll 0$ also yielded flat shapes (Fig. S17). In either case, the aspect ratio and height of the disc is controlled by $\overline{\sigma}$. Lower heights (smaller R_{rim}) were achieved by increasing $\overline{\sigma}$.

Concave adhered discs were fitted with concave top curvature, $\overline{U}_0 = -1$, and underpressure, $\Delta\overline{P} < 0$. Decreasing $\Delta P < 0$ reduced the curvature at the top of the vesicle, U_0, culminating in U_0 for $\Delta P \ll 0$. Similar to the disc-like shapes, R_{rim} could be matched to observations by adjusting $\overline{\sigma}$. Importantly, this fitting method works on adhered shapes that show impact from bending on the shape. It fails when fitting spherical caps in the strong adhesion limit, where the bending does not impact the shape, which is needed for correct scaling of the energies. Furthermore, for $\Delta P < 0$ and $\Delta\overline{P}/\overline{\sigma} = -(2 + \varepsilon)$, the model yields unphysical predictions where the top of the membrane penetrates the adhered portion of the vesicle.

To obtain numerical predictions for $-0.25 < C_{top}R_0 < 0.25$ (Figs. 5F and 6C-F), the shape equations were solved using the shooting method from the top of the vesicle and by

setting as initial condition $U_0 = C_{top} = 0$. The unknown value of $\sigma R_0^2/\kappa$ was used to satisfy the total area constraint, $A/(4\pi R_0^2) = 1$, for some fixed $\Delta P R_0^3/\kappa$. This gives a shape for $C_{top} = 0$ with a certain reduced volume. This was then repeated over a range of values of $\Delta P R_0^3/\kappa$, yielding a family of shapes with $C_{top} = 0$ and possessing various reduced volumes. The same process was used to obtain shapes with $C_{top} R_0 = -0.25$ and $C_{top} R_0 = 0.25$.

Quantification of Shape Geometries

The vesicle area was calculated by integrating $X(s)$ from the best-fit shape,

$$A = \int_0^{s_1} 2\pi X \, \mathrm{d}s \tag{S18}$$

and the volume was determined using

$$V = \int_0^{s_1} 2\pi X^2 \sin(\psi) \, \mathrm{d}s \, . \tag{S19}$$

The purely geometric quantities (Figs. 3 and 4), including vesicle area, volume, reduced volume, height, maximum radius, and vesicle top radius of curvature were obtained for all vesicles (including spherical caps where shape analysis was not feasible) using the manual contour tracking method previously reported by Steinkühler et. al.[10]

The reduced volume ν was calculated using

$$\nu = \frac{V}{V_0} = \frac{V}{\frac{4\pi}{3} \cdot R_0^3} = 6\sqrt{\pi} \frac{V}{A^{3/2}}, \tag{S20}$$

where V is the volume of the deflated object, V_0 is the volume of a sphere with the same surface area as the object. Therefore, lower reduced volumes indicate a higher degree of deflation or respectively a larger surface-to-volume ratio. The reduced volume of a sphere is $\nu_0 = 1$.

Bendocapillary Length and Rim Height

The relationship $R_{rim} \approx \lambda_\kappa$ with $\lambda_\kappa \equiv \sqrt{\kappa/\sigma}$ the bendocapillary length was derived following Forêt et al.[11] Briefly, the shape of the highly curved rim of an axisymmetric vesicle is well-described by the shape of a 2-dimensional contour that extends infinitely into the plane of the contour. This approximation holds as long as the equatorial curvature along the widest part of the vesicle, $C_2 = \sin\psi/X$, which is at most of order $O(1/R_0)$, is negligible relative to the rim curvature $C_1 = \dot{\psi}$, which is of order $O(1/\lambda_\kappa)$. The rim of a flat disc-like vesicle then forms syntractrix given by[11]

$$x(z) = \lambda_\kappa \ln\left(\frac{2\lambda_\kappa + \sqrt{4\lambda_\kappa^2 - z^2}}{z}\right) - \sqrt{4\lambda_\kappa^2 - z^2}, \tag{S21}$$

which is defined for $0 < z \leq 2\lambda_\kappa$. In particular, this z-range implies that $h = 2\lambda_\kappa$ and therefore that $R_{rim} = h/2 = \lambda_\kappa$.

Estimation of PEG-induced Spontaneous Curvature

All-atom molecular dynamics simulations have predicted weak binding of PEG to lipid head-groups with interaction energy of $1.6\,\mathrm{k_B T}$ per PEG molecule, and that this binding is, for the most part, mediated by the PEG's OH end-groups, whose concentration is twice the molar concentration of PEG.[12] Furthermore, this binding was shown to induce significant spontaneous curvature in giant vesicles containing approximately 3.5% (w/v) (4 mM) PEG 8 kDa (and no PEG outside).[12] To estimate the magnitude of PEG-induced spontaneous curvature, C_0, in our system, we also must consider the larger size of PEG 100 kDa compared to PEG 8 kDa using

$$C_0 \approx \frac{\mathrm{RT}c}{4\kappa R_g \mathrm{K}_d}, \tag{S22}$$

where c is the molar PEG concentration, R is the gas constant, T is the absolute temperature, R_g is the radius of gyration of PEG, and K_d is the dissociation constant of PEG-membrane

interaction.[13] This equation holds for $c < K_d$, which is the case here. We can estimate $R_g = 3.7$ nm for PEG 8 kDa and $R_g = 15.9$ nm for PEG 100 kDa using scaling reported by Devanand et al.[14] For PEG 8 kDa, we use $K_d = 200$ mM based on the membrane-binding energy of 1.6 k_BT per PEG molecule. Since both PEG 8 kDa and PEG 100 kDa have two OH-groups per molecule, we can assume that both polymers have the same K_d. We can then estimate the ratio of spontaneous curvature induced by PEG 8 kDa to that of PEG 100 kDa:

$$\frac{C_{0,8K}}{C_{0,100K}} \approx \frac{c_{8K}}{c_{100K}} \cdot \frac{R_{g,100K}}{R_{g,8K}} \approx 200. \tag{S23}$$

In other words, we expect the effect of PEG-induced spontaneous curvature to be at least two orders of magnitude smaller than under the conditions described in Liu et al., explaining why, even with 0.8% PEG 100 kDa, we don't see significant spontaneous curvature. We therefore conclude that our membranes are well-described by a model with zero spontaneous curvature and that the derived physical parameters are valid.

Adhesion Between Golgi Cisternae

The spontaneous curvature model predicts that a normalized adhesion strength of $\tilde{\omega} \approx$ 1,400 to a flat rigid substrate would be required to shape a vesicle with $R_0 \approx 350$ nm and reduced volume $\nu \approx 0.1$ (and zero spontaneous curvature) into a perfectly flat disc with dimensions of a Golgi cisterna. Using $\kappa \approx 80\,k_BT$ reported for Golgi membranes,[15] yields $\omega \approx 900{,}000\,k_BT/\mu m^2$. This large value corresponds to an idealized *cis*-Golgi cisterna that is perfectly flat on one side and adhered to a rigid flat stack on the other side. If one allows for slight concavity and a non-rigid contact zone, much smaller adhesion strengths could suffice to generate a stack of approximately flat cisternae. Svetina and colleagues have theoretically studied the shapes of stacked lipid membrane compartments in the context of red blood cell rouleau formation.[16–18] They found that at sufficiently small reduced volumes, flat contact zones between vesicles become unstable and instead become sigmoidal. Although it is difficult

to assess the exact situation for reduced volumes $\nu \approx 0.1$ from the published plots,[18] it is conceivable that allowing curved contact zones could reduce the required adhesion strength by as much as an order of magnitude.

Golgi stacking has, at least in part, been attributed to bridging interactions via homodimerization of GRASP65 and GRASP55 between neighboring cisternae.[19] The free energy of dimerization has not been measured, but quantitative mass spectrometry yields ∼13,000 copies of GRASP65 and ∼491,000 copies of GRASP55 per HeLa cell.[20] Given that the total surface area of the Golgi is approximately 100 μm^2 (assuming 100 cisternae of $\sim 1\,\mu m^2$ each), this corresponds to ∼5,000 GRASP proteins (or ∼2,500 GRASP dimers) per μm^2. Although the energies of GRASP homodimerization are unknown, using a reasonable value for proteins, $10\,k_B T$, we get a rough estimate for the average adhesion strength that GRASP proteins could mediate between cisternae: $\omega \approx 25{,}000\,k_B T/\mu m^2$. This number suggests that GRASPs may stack and flatten Golgi cisternae as long as some degree of curvature along the contact zone is maintained.

Supplementary Figures

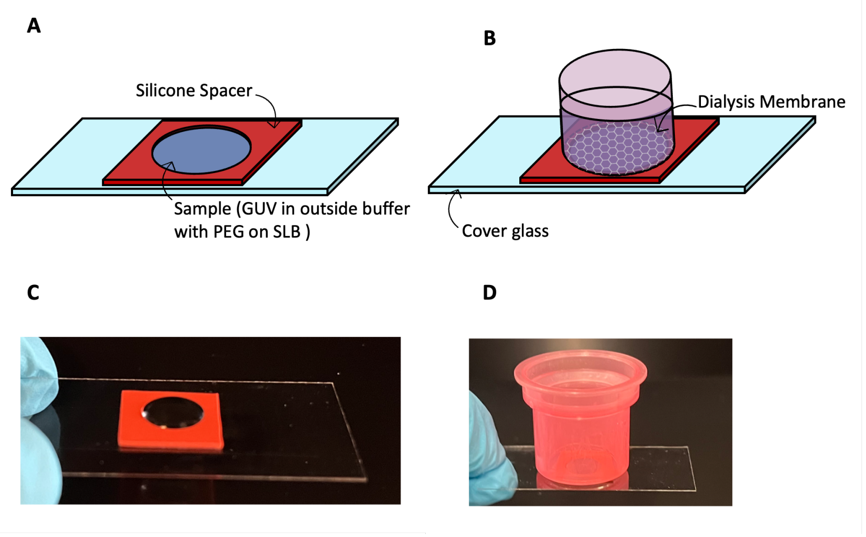

Figure S1: *Diffusion chamber for observation of individual GUVs during successive steps of osmotic deflation* **(A)** Schematic showing the imaging chamber. The supported lipid bilayer (SLB) was generated on the cover glass within the annulus of the silicone spacer. The annulus was then filled with a solution of GUVs in outside buffer containing a fixed concentration of PEG. **(B)** After the GUVs were allowed to sediment, a disposable dialysis cup was filled with deflation buffer and carefully placed on top of the spacer, bringing the dialysis membrane in direct contact with the GUV solution. **(C)** Photograph of the silicone spacer shown in schematic (A). **(D)** Photograph of the diffusion chamber with the dialysis cup placed on top of the silicone spacer, as shown in schematic (B).

Table S1: *Parameters for the best-fit axisymmetric vesicle shapes from Fig. 5.* Columns correspond to vesicles shown in Fig. 5 (A), (B), and (C). The top 4 rows list the dimensionless parameters for numerical integration of the shape equations (Eqs. S9 - S12), where $\Delta\overline{P} = \Delta P \cdot R_{top}^3/\kappa$, $\overline{\sigma} = \sigma \cdot R_{top}^2/\kappa$, and R_{max} is the maximum disc radius. Subsequent rows list the corresponding dimensionful quantities assuming $\kappa = 33\, k_BT$.

	**A**	**B**	**C**
$\Delta\overline{P}$	399.94	0	-1000
$\overline{\sigma}$	200	10^6	484
R_{max} (μm)	11.5	11	11
Curvature direction	convex	convex	concave
R_{top} (μm)	16	1358	30
ω ($k_BT/\mu\text{m}^3$)	48	45	47
σ ($k_BT/\mu\text{m}^2$)	27	18	18
ΔP ($k_BT/\mu\text{m}^3$)	3.5	0	-1.2
A (μm^2)	1018	852	838
A_{adh} (μm^2)	360	338	340
V (μm^3)	1900	926	625
ν	0.64	0.40	0.27
A_{adh}/A	0.35	0.40	0.40

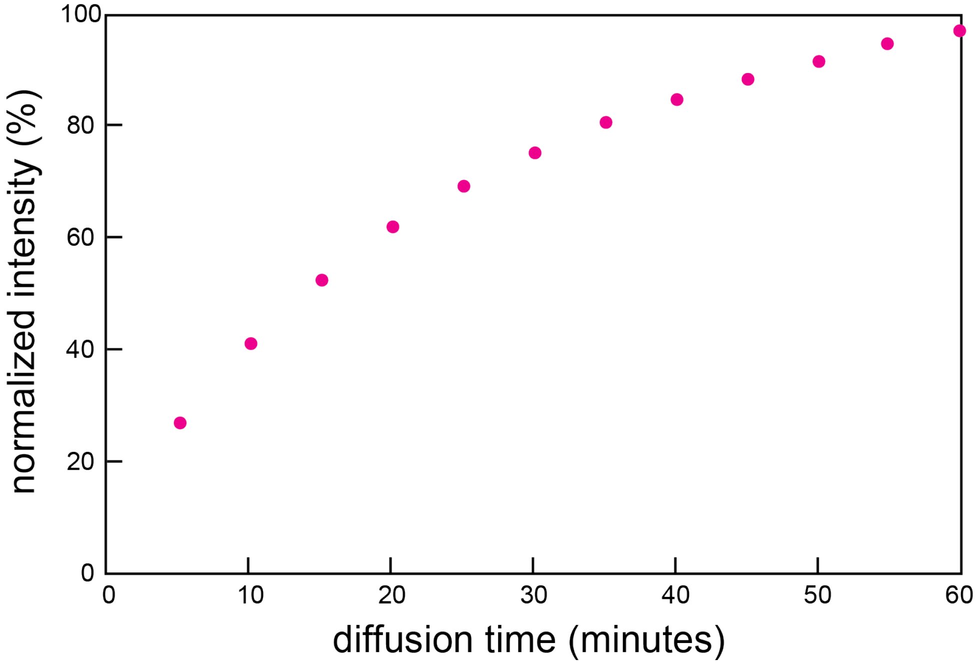

Figure S2: *Equilibration time scale via diffusion.* Time series of normalized fluorescence intensity of Rhodamine B diffusing from the top of the dialysis cup (1.25 g/L final) into the sample chamber. Each data point is the mean intensity from 13 locations immediately above the supported lipid bilayer. The values are normalized to fluorescence intensity measured by addition of 1.25 g/L Rhodamine directly to the bilayer.

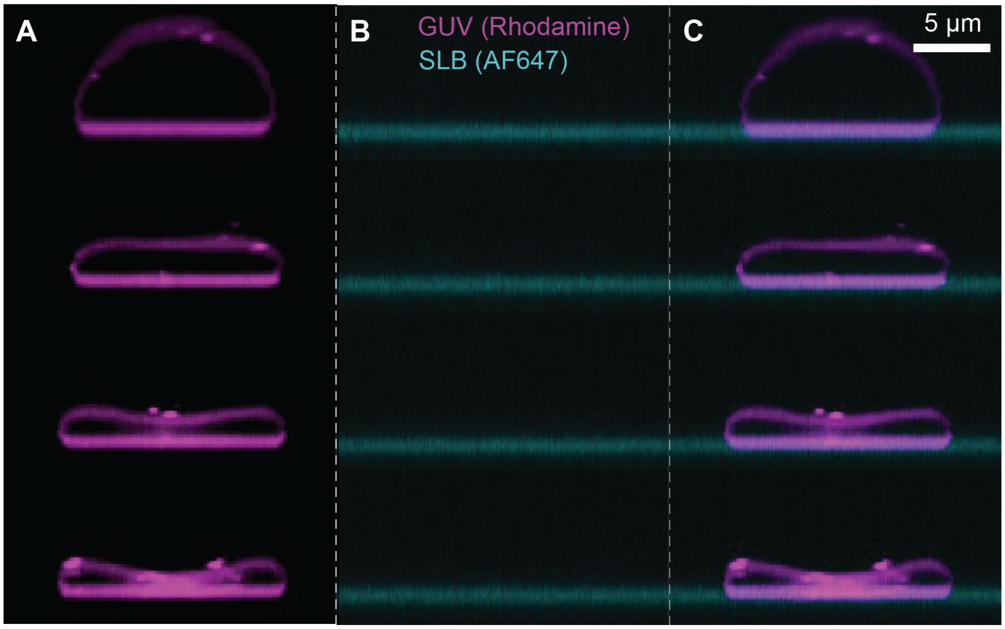

Figure S3: *Two-color confocal imaging of fluorescently labelled lipids in the GUV and supported lipid bilayer (SLB) membranes.* Orthogonal xz cross-sections show a single GUV (*magenta*) on a SLB (*cyan*) undergoing deflation. **(A)** GUVs containing rhodamine-DOPE lipids. **(B)** SLB containing DBCO-DOPE lipids labelled with AlexaFluor647-azide using copper-free click chemistry. **(C)** Overlay of GUV and SLB channels. The SLB and GUV membranes do not exchange lipids over the course of adhesion and deflation, indicating absence of hemi-fusion or full fusion between the two membranes.

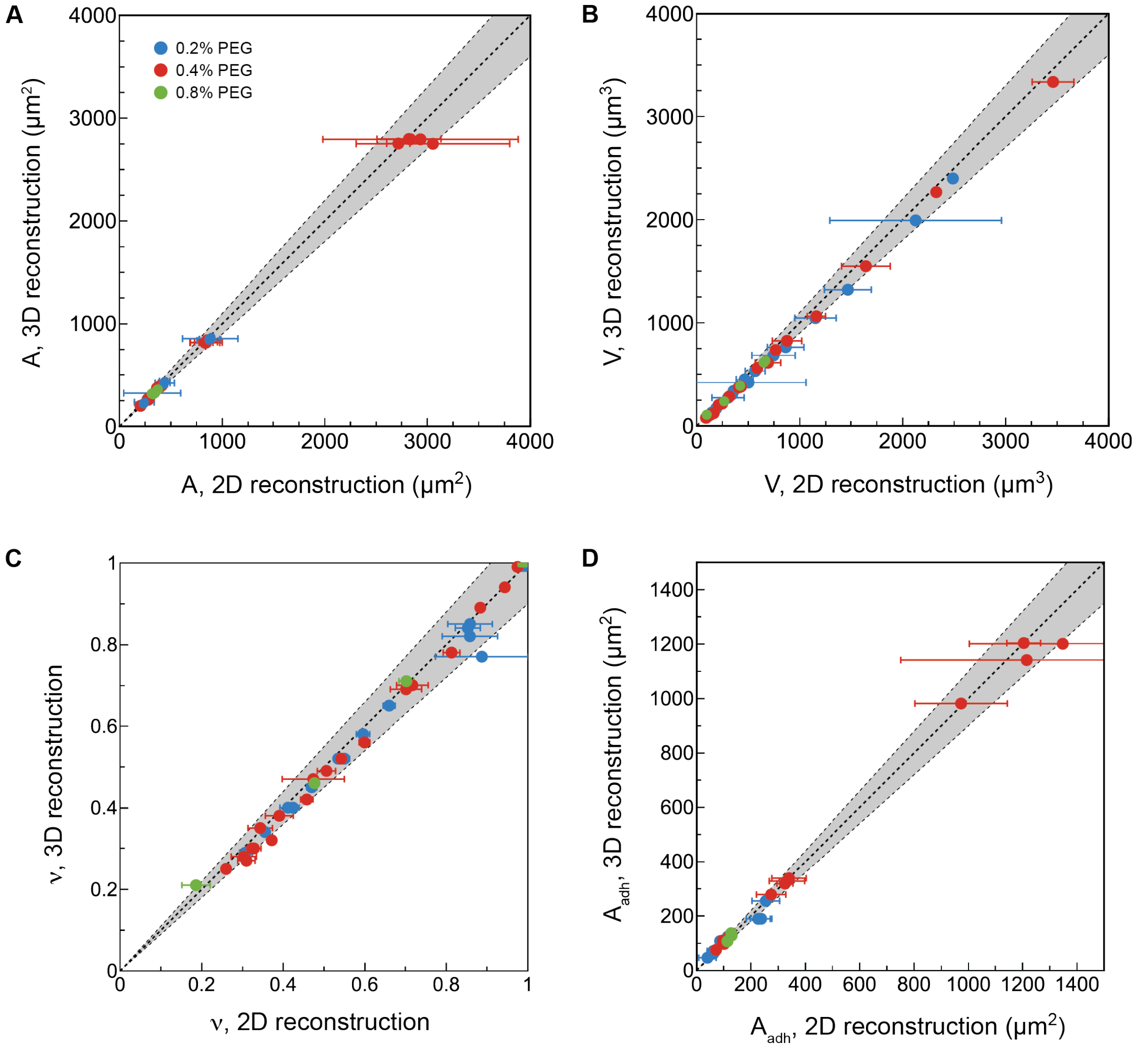

Figure S4: *Comparison between geometric observables obtained from three-dimensional reconstruction and a simplified approach, assuming axiosymmetry and inference from a two-dimensional contour within a radial slice of the vesicle.* The two methods yield the same results within 10% accuracy (gray shaded area) for **(A)** the membrane surface area A, **(B)** the enclosed volume V, **(C)** the reduced volume ν, and **(D)** the adhered area A_{adh}. The error bars represent the standard deviation among measurements from three radial slices of a vesicle. For the 3D reconstruction, a custom python script was used. Colors indicate the concentration of PEG 100 kDa in the outside buffer: 0.2% (w/v) (*blue*), 0.4% (w/v) (*red*), 0.8% (w/v) (*green*).

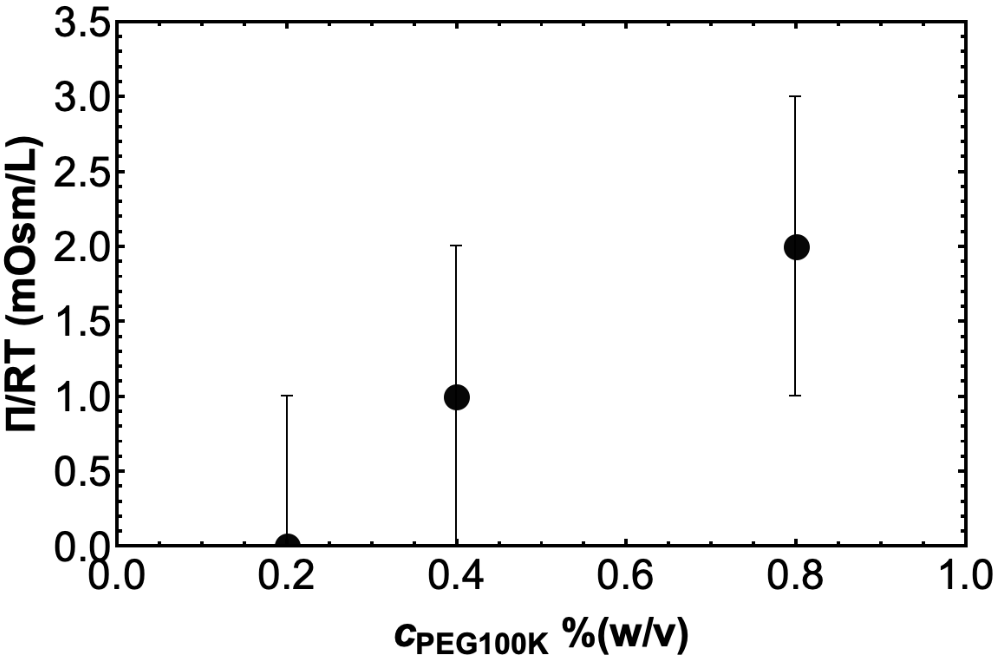

Figure S5: Measured osmotic pressures of 0.2% (w/v), 0.4% (w/v), and 0.8% (w/v) PEG 100 kDa in pure water using freezing point osmometry.

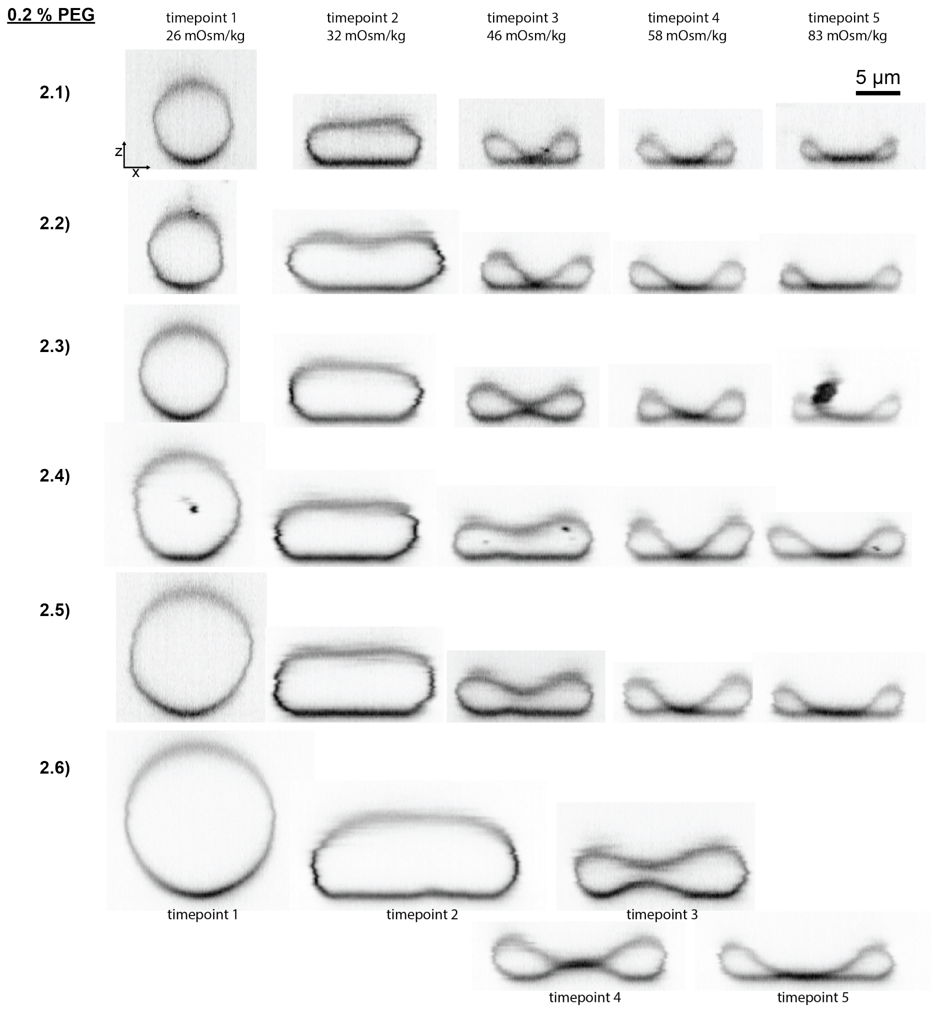

Figure S6: *Radial slices from confocal z-stacks of vesicles at 0.2 % (w/v) PEG sorted by increasing size.* Each row corresponds to one vesicle imaged over the course of the deflation sequence. Scale bar: 5 um.

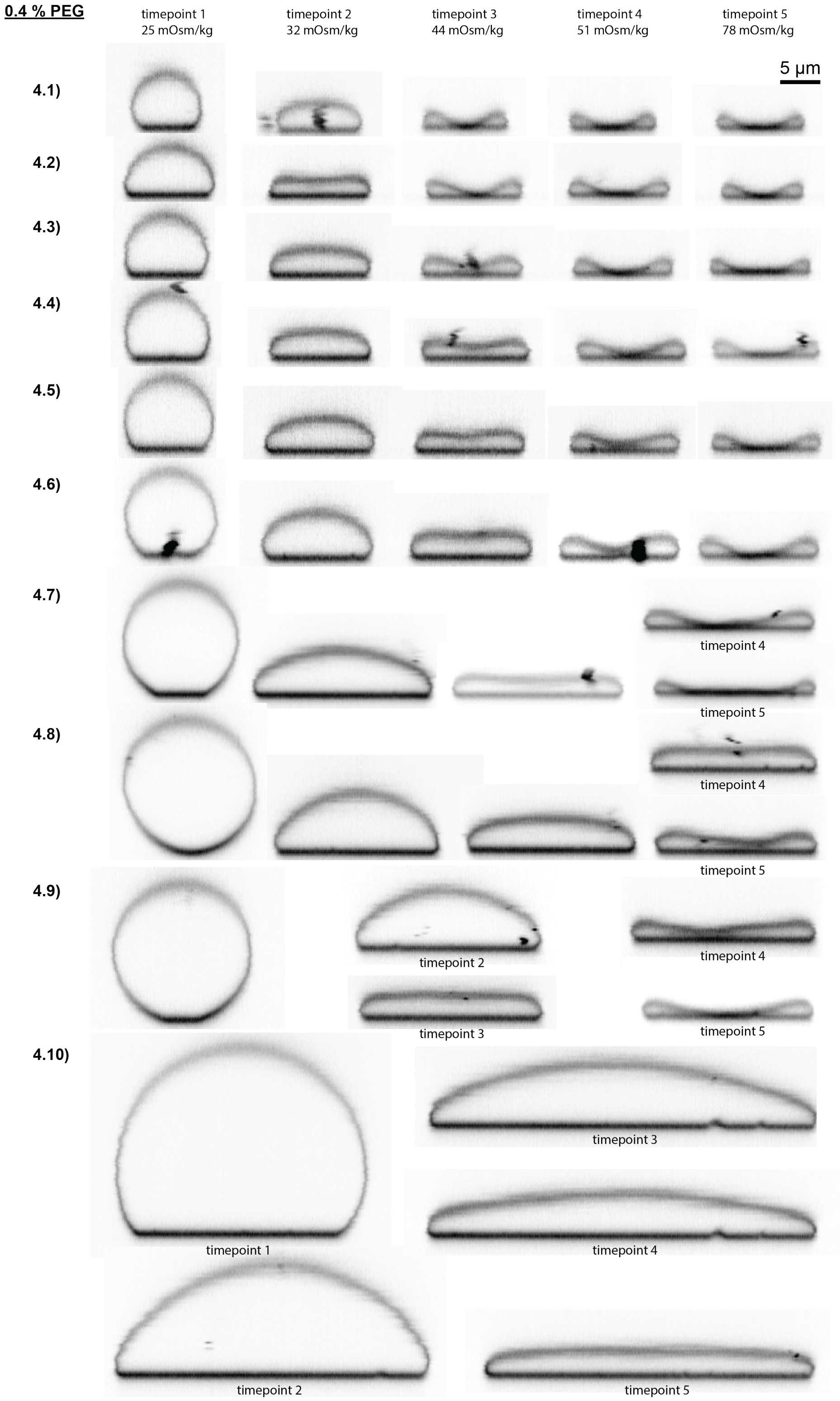

Figure S7: *Radial slices from confocal z-stacks of vesicles at 0.4 % (w/v) PEG sorted by increasing size.* Each row corresponds to one vesicle imaged over the course of the deflation sequence. Scale bar: 5 um.

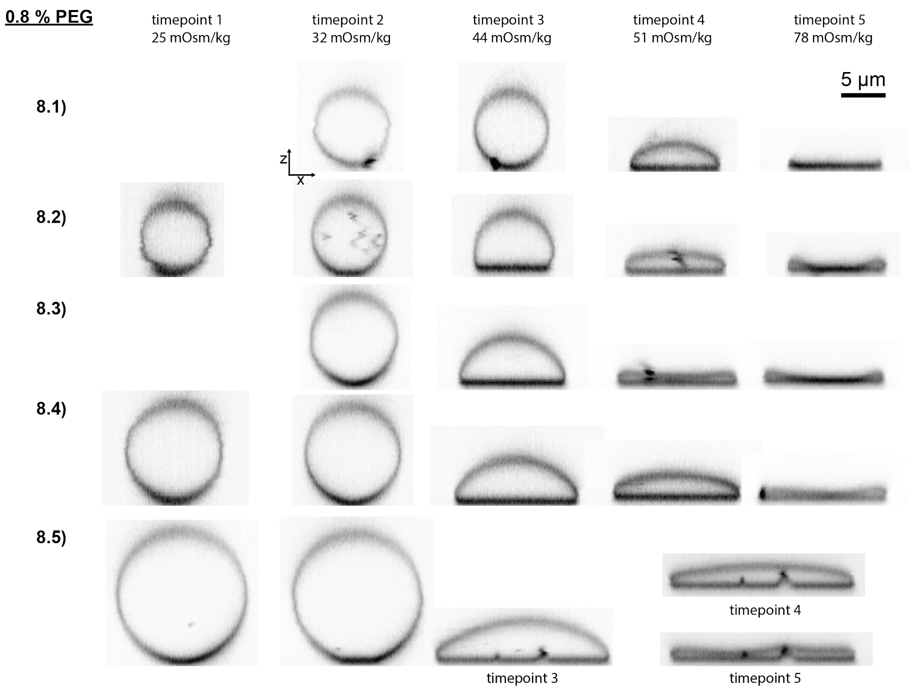

Figure S8: *Radial slices from confocal z-stacks of vesicles at 0.8 % (w/v) PEG sorted by increasing size.* Each row corresponds to one vesicle imaged over the course of the deflation sequence. Scale bar: 5 um.

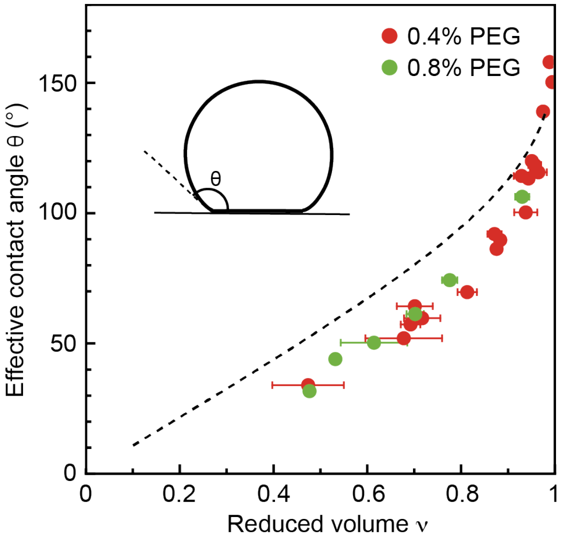

Figure S9: *Effective contact angle of spherical cap-like shapes as a function of reduced volume.* The effective contact angle θ was determined using the ImageJ Angle tool. Black dashed curve denotes the effective contact angle predicted for perfect spherical caps in the limit of infinitely strong adhesion (Eq. 2). The colors indicate the concentration of PEG 100 kDa in the outside buffer: 0.4% (w/v) (*red*) 0.8% (w/v) (*green*). Error bars indicate standard deviations among measurements obtained from 3 different confocal slices (see Methods and Materials for details).

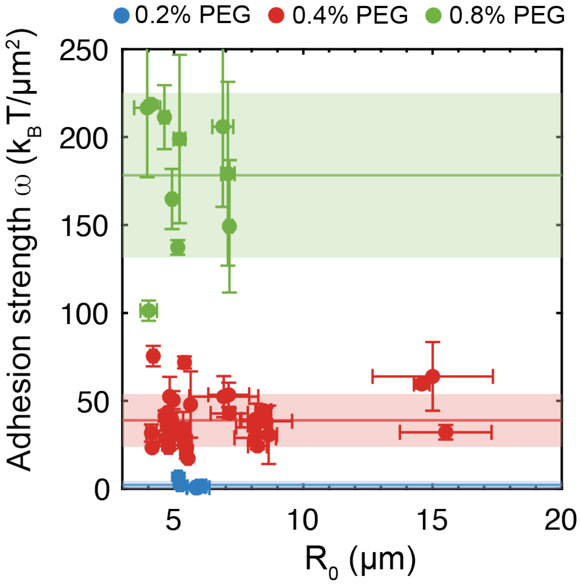

Figure S10: *Adhesion strength as a function of the vesicle size* R_0 The adhesion strength depends on the PEG concentration, but not on the size of the vesicles. This demonstrates the absence or negligible of a spontaneous curvature effect, as there is no vesicle-size dependence of the adhesion strength.

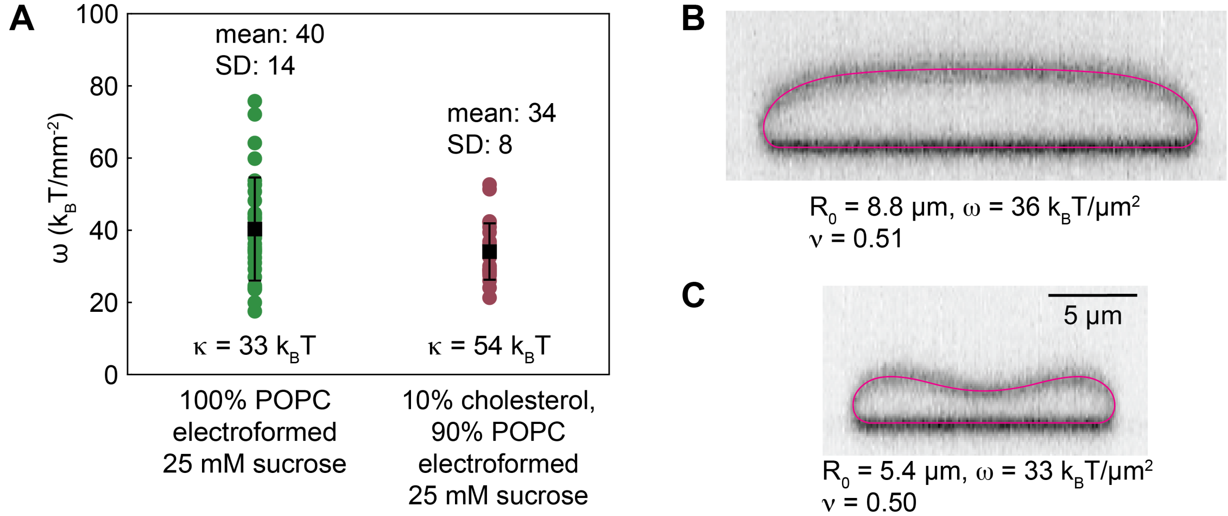

Figure S11: *Shape analysis of two deflated and adhered vesicles undergoing fast lipid flip-flop.* **(A)** Comparison of adhesion strengths in in for cholesterol-free (*green*) and cholesterol-containing (*red*) giant vesicles. Two typical vesicles with 10% cholesterol with two different sizes **(B)** R_0 = 8.8 μm and **(C)** R_0 = 5.4 μm yielded similar adhesion strengths. The vesicles were adhered in the presence of 0.4% PEG 100 kDa and membranes contained 10 mol% cholesterol, 89.9 mol% POPC, and 0.1 mol% Rhodamine-PE. In this data set, only one slice per vesicle time point was analyzed, which is the reason why the standard deviation for 100 mol% POPC is slightly smaller than in Fig. 5D (*red*).

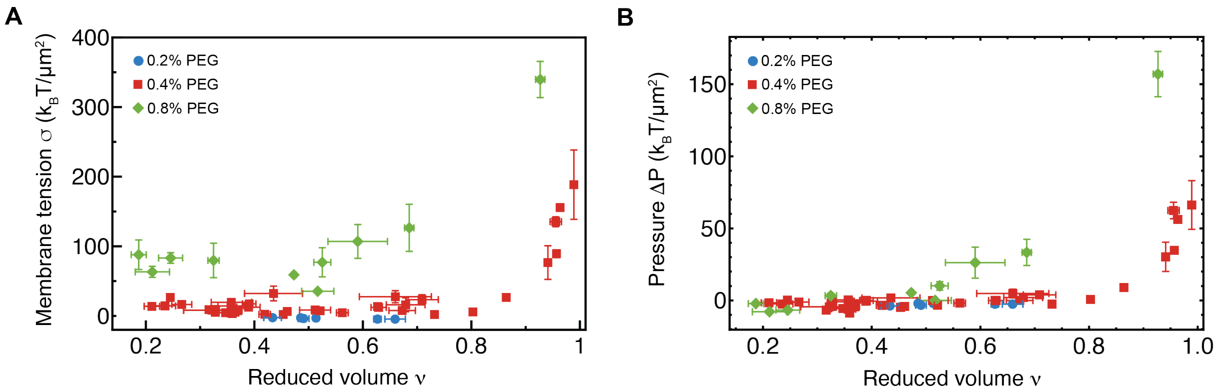

Figure S12: *Membrane tension and pressure from shape analysis* **(A)** Membrane tension, σ, as a function of reduced volume. **(B)** Pressure difference, ΔP, as a function of reduced volume.

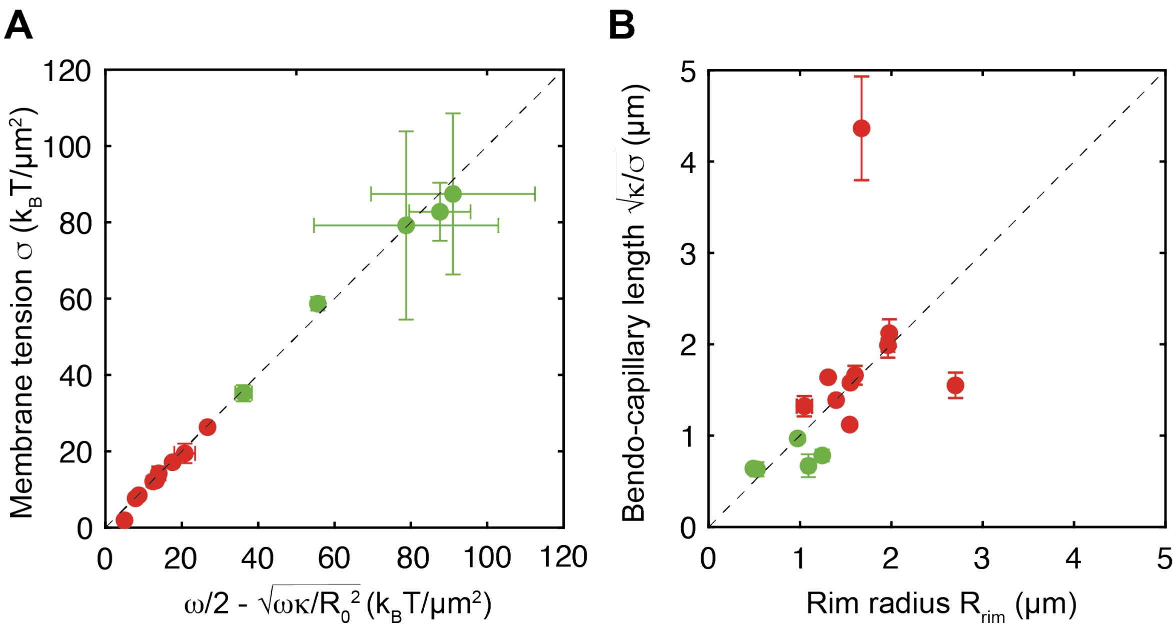

Figure S13: *Variation of Fig. 6 D and E with dimensions.* **(A)** The dimensional modified Young's law sets the tension, addition to Fig. 6D. **(B)** The vesicle height is set by the bendo-capillary length, Fig. 6E.

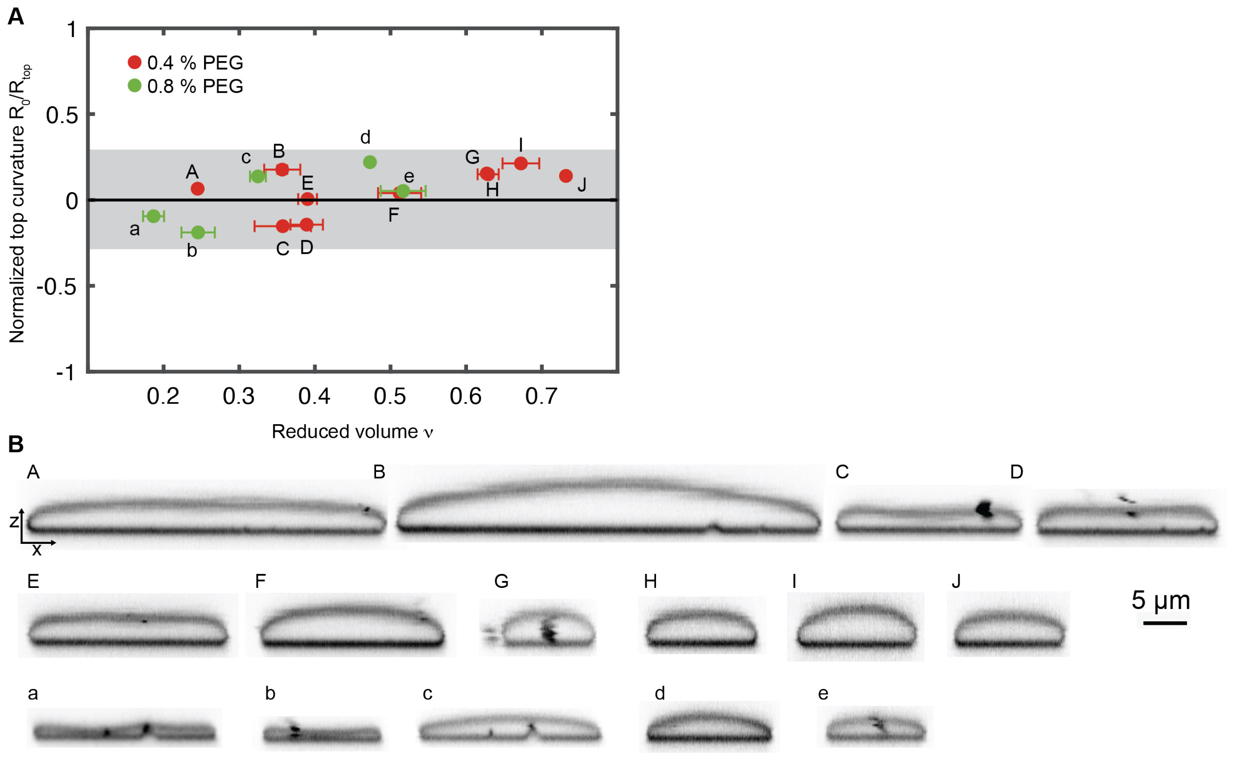

Figure S14: *Overview of flat disc-like vesicles shown in Fig. 6* **(A)** Normalized to top curvatures and reduced volumes of the flat disc-like vesicles along with their labels at 0.4% (w/v) PEG (*capital letters*) and 0.8%v (w/v) PEG (*small letters*). **(B)** radial slices of the flat disc-like vesicles with labels as defined in (A).

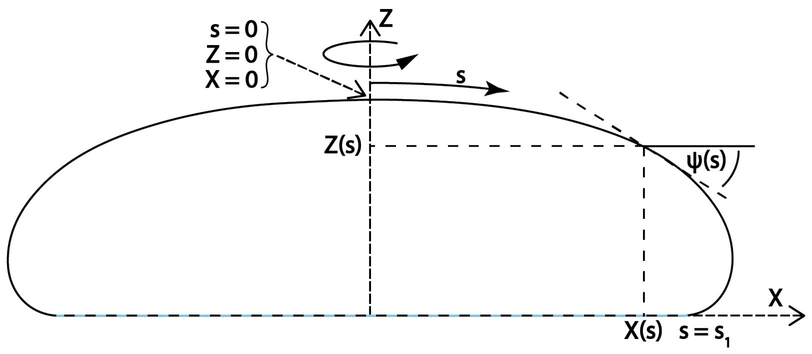

Figure S15: *Parametrization of the adhered vesicle shape used in the modeling.* The shape is an orthogonal xz cross-section, with rotational symmetric around the z axis. The shape is defined by the arclength S and the its slope ψ. The adhered membrane starts at an arclength $S = S_1$ and is marked by a blue-black dashed line.

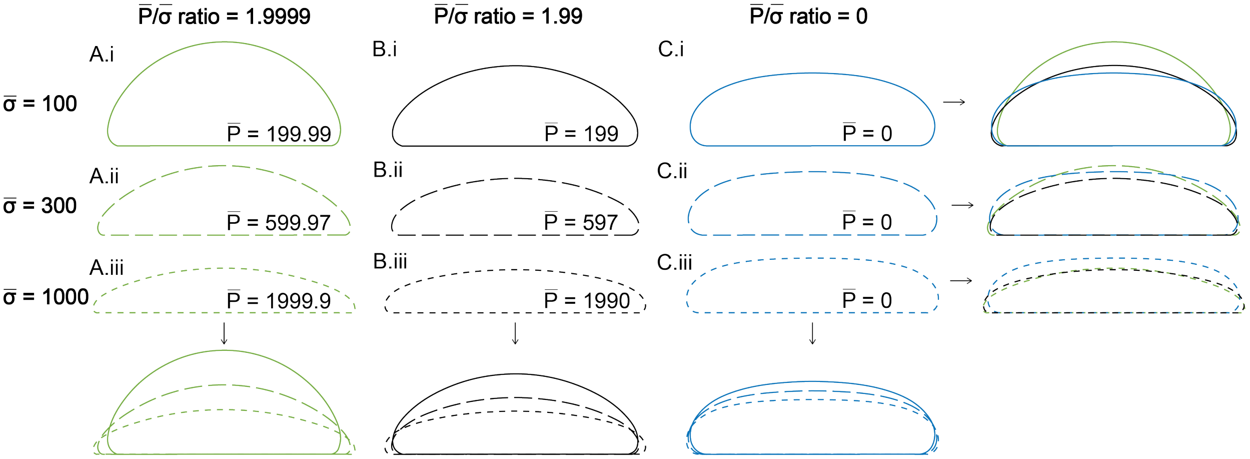

Figure S16: *Convex to disc-like shapes calculated by numerically integrating the shape equations of the Canham-Helfrich model.* Each of the three rows *(i)-(iii)* corresponds to a non-dimensionalized tension $\overline{\sigma} = \sigma \cdot R_{top}^2/\kappa$, with *(i)* $\overline{\sigma} = 100$, *(ii)* $\overline{\sigma} = 300$, and *(iii)* $\overline{\sigma} = 1000$. Each of the three columns *(A)-(C)* corresponds to a particular pressure-to-tension ratio with *(A)* $\overline{P}/\overline{\sigma} = 1.9999$ *(B)* $\overline{P}/\overline{\sigma} = 1.99$, and *C)* $\overline{P}/\overline{\sigma} = 0$. The lowest row and the right-most column show overlaid shapes. Comparing the shapes within a column, reveals that increasing $\overline{\sigma}$ decreases the aspect ratio and and the reduced volume. Comparing the shapes within a row, a decrease in $\Delta\overline{P}/\overline{\sigma}$ decreases increases R_{top} and decreases the curvature at the rim. All vesicles are rescaled to a total surface area of $A = 1000\,\mu\text{m}^2$. Extracted parameters are shown in Table S2.

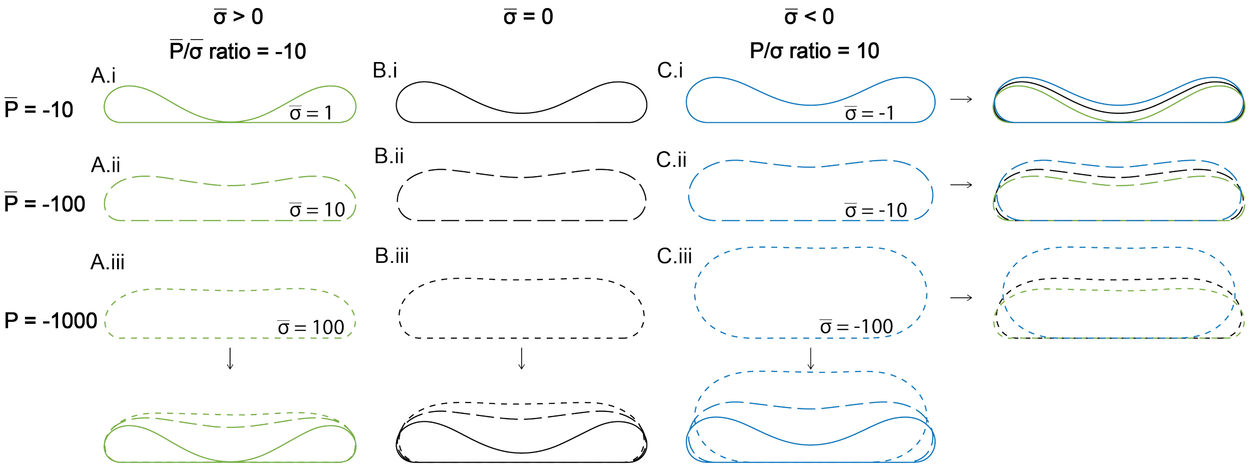

Figure S17: *Concave disc-like vesicle shapes calculated by numerically integrating the shape equations of the Canham-Helfrich model.* Each of the three rows *(i)*-*(iii)* corresponds to a non-dimensionalized pressure difference $\Delta\overline{P} = \Delta P \cdot R_{top}^3/\kappa$, with *(i)* $\Delta\overline{P} = 10$, *(ii)* $\Delta\overline{P} = 100$, and *(iii)* $\Delta\overline{P} = 1000$. Each of the three columns *(a)*-*(c)* corresponds to a particular pressure-to-tension ratio with *(a)* $\overline{P}/\overline{\sigma} = 10$ *(b)* $\overline{P}/\overline{\sigma} = 0$, and *c)* $\overline{P}/\overline{\sigma} = -10$. The lowest row and the right-most column show overlaid shapes. Comparing the shapes within a column, reveals that increasing $\Delta\overline{P}$ decreases R_{top} and decreases the curvature at the rim. Comparing the shapes within a row, a decrease in $\Delta\overline{P}/\overline{\sigma}$ decreases the aspect ratio and increases the reduced volume. All vesicles are rescaled to a total surface area of $A = 1000\,\mu\mathrm{m}^2$. Extracted parameters are shown in Table S3.

Table S2: *Parameters for the calculated shapes in Fig. S16 solving the Canham-Helfrich model.* Columns correspond to vesicles shown in Fig. S16. The top 4 rows list the dimensionless parameters for numerical integration of the shape equations (Eqs. S9 - S12), $\Delta\overline{P} = \Delta P \cdot R_{top}^3/\kappa$, $\overline{\sigma} = \sigma \cdot R_{top}^2/\kappa$, R_{max} is the maximum disc radius, and curvature direction is denoted by "-" for concave and "+" for convex shapes. Subsequent rows list the corresponding dimensionful quantities assuming $\kappa = 33\, k_B T$.

	A.i	A.ii	A.iii	B.i	B.ii	B.iii	C.i	C.ii	C.iii
$\Delta\overline{P}$	199.99	599.97	1999.9	199	597	1990	0	0	0
$\overline{\sigma}$	100	300	1000	100	300	1000	100	300	1000
R_{max} (μm)	10.64	11.52	11.95	11.19	11.29	11.88	11.17	11.35	11.50
Curvature direction	+	+	+	+	+	+	+	+	+
R_{top} (μm)	11.51	16.29	27.42	16.04	23.34	39.48	35.59	52.47	82.49
ω (k_BT/μm^3)	20.50	40.91	55.31	14.97	23.75	29.22	5.51	7.00	8.88
σ (k_BT/μm^2)	14.70	22.02	25.89	7.56	10.72	12.49	1.54	2.12	2.86
ΔP (k_BT/μm^3)	2.56	2.70	1.89	0.94	0.91	0.63	0	0	0
A (μm^2)	1000	1000	1000	1000	1000	1000	1000	1000	1000
A_{adh} (μm^2)	290	373	412	323	347	394	288	312	332
V (μm^3)	2323	1771	1320	2036	1518	1370	2062	1902	1746
ν	0.78	0.60	0.44	0.68	0.56	0.46	0.69	0.64	0.59
A_{adh}/A	0.29	0.37	0.41	0.32	0.37	0.39	0.29	0.31	0.33

Table S3: *Parameters for the calculated shapes in Fig. S17 solving the Canham-Helfrich model.* Columns correspond to vesicles shown in Fig. S17. The top 4 rows list the dimensionless parameters for numerical integration of the shape equations (Eqs. S9 - S12), $\Delta\overline{P} = \Delta P \cdot R_{top}^3/\kappa$, $\overline{\sigma} = \sigma \cdot R_{top}^2/\kappa$, R_{max} is the maximum disc radius, and curvature direction is denoted by "-" for concave and "+" for convex shapes. Subsequent rows list the corresponding dimensionful quantities assuming $\kappa = 33\, k_BT$.

	a.i	a.ii	a.iii	b.i	b.ii	b.iii	c.i	c.ii	c.iii
$\Delta\overline{P}$	10	100	1000	10	100	1000	10	100	1000
$\overline{\sigma}$	1	10	100	0	0	0	-1	-10	-100
R_{max} (μm)	11.53	11.52	11.47	11.48	11.38	11.24	11.40	11.19	10.65
Curvature direction	-	-	-	-	-	-	-	-	-
R_{top} (μm)	7.27	17.87	43.67	7.47	17.95	41.72	7.60	17.71	37.05
ω (k_BT/μm^3)	4.00	4.63	5.35	3.12	3.17	2.97	2.32	1.72	0.09
σ (k_BT/μm^2)	0.37	0.61	1.02	0	0	0	-0.34	-0.62	-1.42
ΔP (k_BT/μm^3)	-0.51	-0.34	-0.23	-0.47	-0.34	-0.27	-0.44	-0.35	-0.38
A (μm^2)	1000	1000	1000	1000	1000	1000	1000	1000	1000
A_{adh} (μm^2)	313	314	312	298	287	271	279	245	87
V (μm^3)	1123	1579	1724	1294	1752	1956	1454	1960	2482
ν	0.38	0.53	0.58	0.44	0.59	0.66	0.49	0.66	0.83
A_{adh}/A	0.31	0.31	0.31	0.30	0.29	0.27	0.28	0.25	0.09

Table S4: *Comparison of in vitro membrane-membrane adhesion systems.* * indicates our estimated values for corresponding system.

**Reference**	ω ($k_BT/\mu m^2$)	ν	**Interface**
This work	2 - 200	0.1 - 1	GUV/SLB with 0.2% - 0.8% PEG 100 kDa
Gruhn et al.[21]	2	0.7 - 0.9	glass
Gruhn et al.[21]	70	0.7 - 0.9	alkanethiolated glass
Steinkühler et al.[22]	0.01 - 0.16	0.7 - 0.9*	ITO-coated glass with electric field
Murakami et al.[23] Chiba et al.[24]	6 - 40	0.6 - 1	GUV doublet/triplet with salt-tuned van der Waals attraction
Evans and Metcalfe[25]	5,000 - 35,000	0.5 - 1*	GUV-GUV micropipette aspiration with 2% - 10% dextran 36.5 kDa
Shimobayashi et al.[26]	150 - 15,000*	0.75 - 0.9*	GUV-SLB adhesion via hybridization of DNA linkers
Amjad et al.[27]	6,000 - 600,000*	0.75 - 0.9*	GUV-SLB adhesion via biotin-streptavidin binding
Indra et al.[28]	100,000*	0.75 - 0.9*	GUV-SLB via cadherins

References

(1) Spanke, H. T.; Style, R. W.; François-Martin, C.; Feofilova, M.; Eisentraut, M.; Kress, H.; Agudo-Canalejo, J.; Dufresne, E. R. Wrapping of microparticles by floppy lipid vesicles. *Physical Review Letters* **2020**, *125*, 198102.

(2) Angelova, M. I.; Dimitrov, D. S. Liposome electroformation. *Faraday discussions of the Chemical Society* **1986**, *81*, 303–311.

(3) Angelova, M. Preparation of giant vesicles by external AC electric fields. Kinetics and application. *Progr. Colloid Polym. Sci. J.* **1992**, *77*, 2090–2101.

(4) Spanke, H. T.; Agudo-Canalejo, J.; Tran, D.; Style, R. W.; Dufresne, E. R. Dynamics of spontaneous wrapping of microparticles by floppy lipid membranes. *Physical Review Research* **2022**, *4*, 023080.

(5) Testa, A.; Spanke, H. T.; Jambon-Puillet, E.; Yasir, M.; Feng, Y.; Küffner, A. M.; Arosio, P.; Dufresne, E. R.; Style, R. W.; Rebane, A. A. Surface Passivation Method for the Super-repellence of Aqueous Macromolecular Condensates. *Langmuir* **2023**, *39*, 14626–14637.

(6) Staykova, M.; Holmes, D. P.; Read, C.; Stone, H. A. Mechanics of surface area regulation in cells examined with confined lipid membranes. *Proceedings of the National Academy of Sciences* **2011**, *108*, 9084–9088.

(7) Testa, A.; Dindo, M.; Rebane, A. A.; Nasouri, B.; Style, R. W.; Golestanian, R.; Dufresne, E. R.; Laurino, P. Sustained enzymatic activity and flow in crowded protein droplets. *Nature Communications* **2021**, *12*.

(8) Seifert, U.; Berndl, K.; Lipowsky, R. Shape transformations of vesicles: Phase diagram for spontaneous-curvature and bilayer-coupling models. *Physical review A* **1991**, *44*, 1182.

(9) Seifert, U.; Lipowsky, R. Adhesion of vesicles. *Physical Review A* **1990**, *42*, 4768.

(10) Steinkühler, J.; Knorr, R. L.; Zhao, Z.; Bhatia, T.; Bartelt, S. M.; Wegner, S.; Dimova, R.; Lipowsky, R. Controlled division of cell-sized vesicles by low densities of membrane-bound proteins. *Nature communications* **2020**, *11*, 905.

(11) Forêt, L. Shape and energy of a membrane bud induced by protein coats or viral protein assembly. *The European Physical Journal E* **2014**, *37*, 1–13.

(12) Liu, Y.; Agudo-Canalejo, J.; Grafmüller, A.; Dimova, R.; Lipowsky, R. Patterns of Flexible Nanotubes Formed by Liquid-Ordered and Liquid-Disordered Membranes. *ACS nano* **2016**, *10 1*, 463–74.

(13) Lipowsky, R. Spontaneous tubulation of membranes and vesicles reveals membrane tension generated by spontaneous curvature. *Faraday discussions* **2013**, *161*, 305–31; discussion 419–59.

(14) Devanand, K.; Selser, J. C. Asymptotic behavior and long-range interactions in aqueous solutions of poly(ethylene oxide). *Macromolecules* **1991**, *24*, 5943–5947.

(15) Upadhyaya, A.; Sheetz, M. P. Tension in tubulovesicular networks of Golgi and endoplasmic reticulum membranes. *Biophysical journal* **2004**, *86*, 2923–2928.

(16) Derganc, J.; Božič, B.; Svetina, S.; Žekš, B. Equilibrium shapes of erythrocytes in rouleau formation. *Biophysical journal* **2003**, *84 3*, 1486–92.

(17) Ziherl, P.; Svetina, S. Flat and sigmoidally curved contact zones in vesicle–vesicle adhesion. *Proceedings of the National Academy of Sciences* **2007**, *104*, 761 – 765.

(18) Svetina, S.; Ziherl, P. Morphology of small aggregates of red blood cells. *Bioelectrochemistry* **2008**, *73 2*, 84–91.

(19) Zhang, X.; Wang, Y. GRASPs in Golgi Structure and Function. *Frontiers in Cell and Developmental Biology* **2016**, *3*.

(20) Itzhak, D. N.; Tyanova, S.; Cox, J.; Borner, G. H. Global, quantitative and dynamic mapping of protein subcellular localization. *elife* **2016**, *5*, e16950.

(21) Gruhn, T.; Franke, T.; Dimova, R.; Lipowsky, R. Novel method for measuring the adhesion energy of vesicles. *Langmuir* **2007**, *23*, 5423–5429.

(22) Steinkühler, J.; Agudo-Canalejo, J.; Lipowsky, R.; Dimova, R. Modulating vesicle adhesion by electric fields. *Biophysical journal* **2016**, *111*, 1454–1464.

(23) Murakami, K.; Ebihara, R.; Kono, T.; Chiba, T.; Sakuma, Y.; Ziherl, P.; Imai, M. Morphologies of vesicle doublets: competition among bending elasticity, surface tension, and adhesion. *Biophysical Journal* **2020**, *119*, 1735–1748.

(24) Chiba, T.; Sakuma, Y.; Imai, M.; Ziherl, P. Morphology of vesicle triplets: shape transformation at weak and strong adhesion limits. *Soft Matter* **2023**, *19*, 4286–4296.

(25) Evans, E.; Metcalfe, M. Free energy potential for aggregation of giant, neutral lipid bilayer vesicles by Van der Waals attraction. *Biophysical journal* **1984**, *46*, 423–426.

(26) Shimobayashi, S. F.; Mognetti, B. M.; Parolini, L.; Orsi, D.; Cicuta, P.; Michele, L. D. Direct measurement of DNA-mediated adhesion between lipid bilayers. *Physical chemistry chemical physics : PCCP* **2015**, *17 24*, 15615–28.

(27) Amjad, O. O.; Mognetti, B. M.; Cicuta, P.; Michele, L. D. Membrane Adhesion through Bridging by Multimeric Ligands. *Langmuir : the ACS journal of surfaces and colloids* **2017**, *33 5*, 1139–1146.

(28) Indra, I.; Choi, J.; Chen, C.-S.; Troyanovsky, R. B.; Shapiro, L.; Honig, B.; Troyanovsky, S. M. Spatial and temporal organization of cadherin in punctate adherens junctions. *Proceedings of the National Academy of Sciences* **2018**, *115*, E4406 – E4415.
